# A Rare Case of Cleidocranial Dysplasia Causing Unilateral Lung Herniation in the Setting of an Acute Viral Infection

**DOI:** 10.7759/cureus.63223

**Published:** 2024-06-26

**Authors:** Mona Ghias, Kevin Bogdansky, Dana Murray, Lindsay Sunzeri, Casandra Arevalo Marcano

**Affiliations:** 1 Internal Medicine, West Virginia University, Morgantown, USA; 2 Nephrology, West Virginia University School of Medicine, Morgantown, USA; 3 Internal Medicine, West Virginia University School of Medicine, Morgantown, USA; 4 Pulmonary Medicine, West Virginia University School of Medicine, Morgantown, USA

**Keywords:** skeletal anomalies, cbfa1, clavicular hypoplasia, respiratory distress, pediatrics & neonatology, lung herniation, cleidocranial dysplasia

## Abstract

Cleidocranial dysplasia (CCD) is a skeletal disorder with potential respiratory complications. We report a case of a 77-day-old male child with CCD who presents in respiratory distress. The infant was found to have a unilateral lung herniation secondary to an acute viral illness. This case highlights the importance of keeping CCD in the differential diagnosis of a neonate in respiratory distress.

## Introduction

Cleidocranial dysplasia (CCD) is an uncommon clinical condition inherited in an autosomal dominant pattern affecting either sex or can occur sporadically. Features of CCD include a triad of dental abnormalities, partial or complete absence of the clavicles, and open sagittal sutures and fontanelles [1]. CCD usually presents as a generalized skeletal dysplasia. The major affected bones are those which undergo intramembranous ossification such as the cranial vault, clavicles, maxilla, nasal, and lacrimal bones [2]. It is characterized by the absence of the clavicles, which usually occurs in 10% of cases or the presence of hypoplastic clavicles which allow the hypermobility of shoulders [2]. Although respiratory problems have rarely been reported in newborns with CCD, thoracic deformity has been frequently described in these patients. Subsequently, a hypoplastic chest can be known to cause respiratory failure in patients with CCD [3]. We present a case of an infant with CCD who developed unilateral lung herniation in the setting of acute viral upper respiratory infection.

## Case presentation

**Figure 1:**
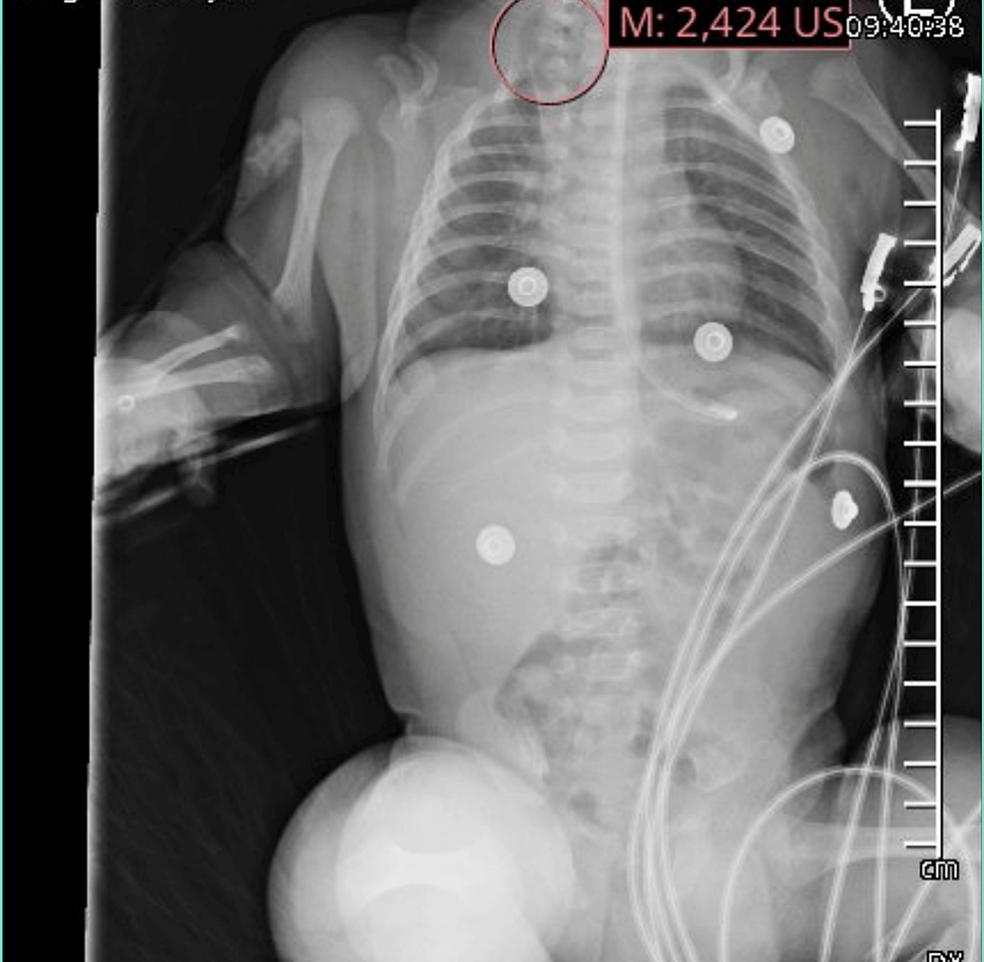
Chest and abdomen X-ray Diffuse interstitial opacities with possible right lung herniation at the thoracic inlet.

**Figure 2:**
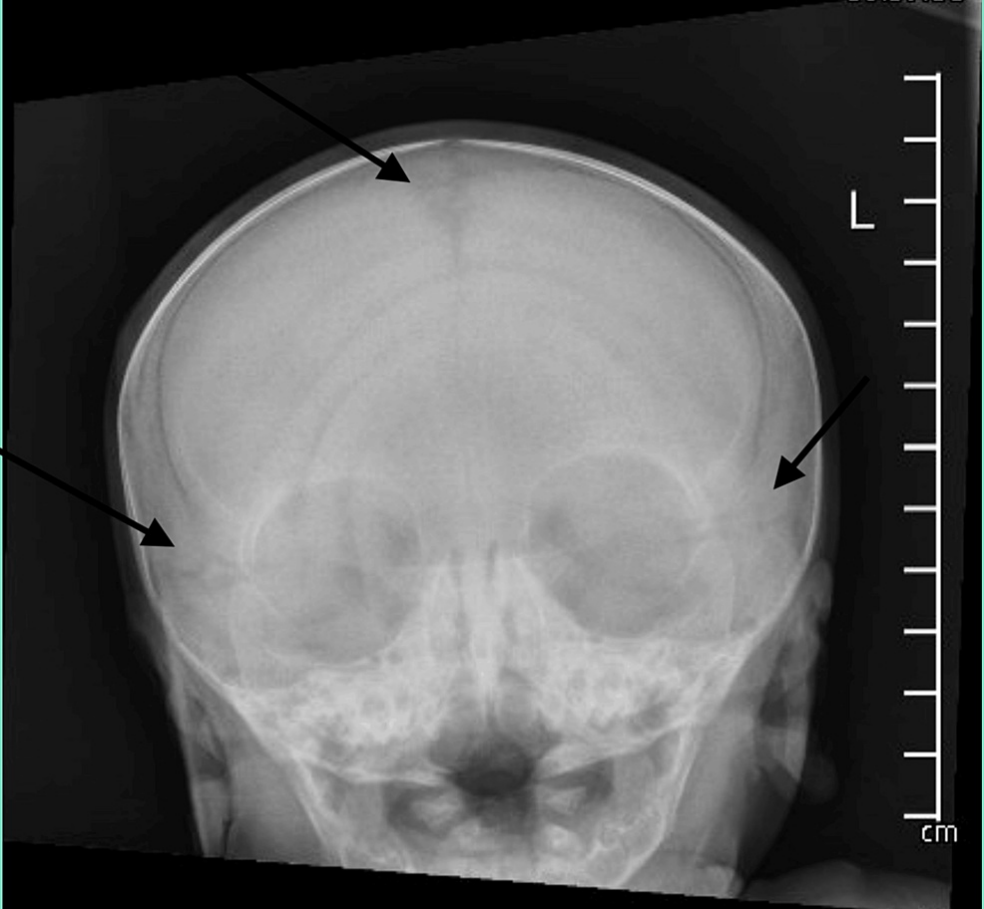
Skeletal survey X-ray Wormian bones are seen at the confluence of the lambdoid and sagittal sutures.

A 77-day-old male child presented with a two-day history of nasal congestion, coughing, poor oral intake, and difficulty breathing in the setting of a viral infection. The infant was born at 35 weeks and 5 days to G2P2 mother via C-section. Fetal ultrasound (US) showed suspicion of coarctation of the aorta; however, a post-natal echocardiogram showed narrowing of the aortic isthmus without significant gradient with a closed ductus arteriosus. During the neonatal intensive care stay, an initial chest X-ray suspected hypoplasia of the right clavicle but no herniation of lung tissue was noted. Physical exam on arrival was relevant for coarse breath sounds and increased accessory muscle use. A chest X-ray (Figure 1) was obtained which revealed lucency in the right thoracic inlet, possibly representing a congenital lung hernia. A skeletal survey (Figure 2) was performed which showed wormian bones seen at the confluence of the lambdoid and sagittal cranial sutures with decreased bone density throughout.

Pediatric pulmonology was consulted during admission and the infant was confirmed to have congenital unilateral partial absence of the clavicles leading to congenital right lung hernia in the thoracic inlet during acute illness. Subsequently, follow-up imaging performed in the outpatient setting after discharge showed resolution of the herniated lung tissue.

## Discussion

CCD, also known as Marie-Sainton disease, is caused by a mutation in the CBFA1 gene [2] and is characterized by multiple skeletal abnormalities. A genetic transition cannot be identified in 40% of the patients and develops spontaneously. The absence of clavicles or incomplete formation of clavicles is characteristic of this syndrome which can be confirmed with a chest X-ray [3,4]. Frontal bossing is usually seen in patients secondary to the delayed closure of the anterior fontanelle and metopic sutures. Abnormal dentition may also be present, including delayed eruption of secondary teeth, retention of primary teeth, and supernumerary teeth [5]. CCD may lead to scoliosis, kyphosis, and other orthopedic abnormalities secondary to skeletal dysplasia. Therefore, there is a high likelihood that patients with CCD will require surgical intervention. With intervention patients with CCD have normal life expectancy [6].

Other conditions that should be considered in the differential for patients with suspected CCD include Crane-Heise syndrome and CDAGS syndrome. Crane-Heise syndrome is a fatal condition with features including hypoplastic clavicles, absence of cervical vertebrae, cleft lip/palate, and multiple cranial and limb anomalies. Similarly to patients with CCD, patients with CDAGS syndrome have delayed closure of fontanelles and hypoplastic clavicles. CDAGS syndrome can be differentiated from CCD by the presence of anal and genitourinary anomalies [5].

Previous cases of CCD have been described in the neonatal period; however, respiratory distress is rarely a presenting symptom. Diagnosis is usually suspected in childhood based on clinical and radiographic features described above and confirmed by genetic testing. The most commonly mutated gene in patients with CCD is RUNX2, which may be detected using single-gene testing, multi-gene panel, and karyotyping [5]. This case shows us that lung herniation is a potential complication that can lead to worsening respiratory status.

## Conclusions

This case illustrates an unusual presentation of CCD. The absence of clavicles due to CCD predisposed this infant to unilateral lung herniation in the setting of acute viral illness. Skeletal anomalies, and specifically clavicular hypoplasia, should be considered in the differential diagnosis of a neonate who presents in respiratory distress. Recognition of CCD and early intervention for respiratory complications, when they happen, are vital for optimal outcomes. Further research is indicated to understand the predisposing factors and the best approach to lung herniation in neonates with CCD.
